# Knowledge, perceptions and effects of Ebola virus disease outbreak on the pig value chain in the agro-pastoralist district of Luwero, Central Uganda

**DOI:** 10.1186/s12879-021-06337-8

**Published:** 2021-07-09

**Authors:** Doreen Birungi, Gabriel Tumwine, Charles Drago Kato, Tonny Ssekamatte, Michael Ocaido, Samuel Majalija

**Affiliations:** 1 School of Biosecurity, Ecosystem health and Veterinary Public Health, College of Veterinary Medicine Animal Resources and Biosecurity, Makerere University, P.O Box 7062, Kampala, Uganda; 2 School of Public Health, College of Health Sciences, Makerere University, P.O Box 7062, Kampala, Uganda; 3 School of Veterinary Medicine and Animal Resources, College of Veterinary Medicine Animal Resources and Biosecurity, Makerere University, P.O Box 7062, Kampala, Uganda

**Keywords:** Ebola, Small holder farms, Food security, Traditional healers, Pig sales

## Abstract

**Background:**

Ebola Virus Disease (EVD) outbreaks have a significant impact on the health and wellbeing, and livelihoods of communities. EVD response interventions particularly affect the food value chain, and income security of pig farmers in agro-pastoral communities. Despite the enormous effort of EVD response interventions, there is paucity of information towards EVD among those involved in the pig value chain, as well as the effect of EVD outbreaks on the pig value chain. This study therefore, assessed the knowledge, perceptions on the occurrence of Ebola and its effects on the pig value chain in the agro-pastoral district of Luweero, Central Uganda.

**Methods:**

A cross sectional study was conducted in two parishes of Ssambwe and Ngalonkulu, Luwero district. A total of 229 respondents were included in the study. Structured questionnaires, key informant interviews and focus group discussions were conducted to collect data. Quantitative data was analysed using SPSS version 22 while qualitative data was analysed using thematic content analysis.

**Results:**

Of the 229 respondents, 95.6% could recall the occurrence of the last EVD outbreak in their locality. About 24.5% associated EVD with touching pigs or eating pork. Regarding knowledge, 194 (84.7%) correctly associated EVD with handling Ebola infected persons, 191 (83.4%) with migration of people from endemic areas, 148 (64.9%) eating monkey meat, 127 (55.5%) with eating bats, and 198 (64.9%) with conducting public meetings where there is an Ebola infected person. Out of 142 farmers, 55 (38.7%) believed that Ebola outbreaks affected demand and sale of pigs. The EVD outbreak significantly led to a reduction in the average number of pigs sold (*P* = 0.001), the average number of pigs bought by traders (*P* = 0.04), and the number of pigs sold/ slaughtered by butcher men at pork eating places (*P* = 0.03).

**Conclusion:**

This study showed that EVD outbreak negatively affected the pig value chain i.e., the demand and supply of pigs and pork. Therefore, there is need to sensitize the stakeholders in the pig value chain on EVD in order to minimize the negative economic impacts associated with EVD outbreaks.

**Supplementary Information:**

The online version contains supplementary material available at 10.1186/s12879-021-06337-8.

## Background

Ebola Virus Disease (EVD) remains a global health challenge. Since the first occurrence, 44 years ago in the Democratic Republic of Congo (DRC) (formerly Zaire), over 38 Ebola outbreaks have been reported [1]. Ebolavirus belongs to the filovirus in the family Filoviridae which consists of four species that cause human illness; *Zaire ebolavirus*, *Bundibugyo ebolavirus*, *Sudan ebolavirus* and *Taï Forest ebolavirus* (also known as *Cote d’Ivoire ebolavirus*) [2]. While *Reston ebolavirus* is only known to cause infection in domestic pigs [3, 4] and possibly pigs could amplify this virus and potentially transmit it to humans in future. EVD seropositive samples have been detected from bats, making them the most probable reservoir species of virus, although no virus has been isolated [5]. Similarly, viral antibodies have been identified in gorillas, chimpanzees, and duikers as other possible sources of infection [5, 6].

EVD outbreaks have occurred in sub-Sahara Africa, largely in the West African countries of Guinea, Liberia, Senegal, Sierra Leone and Nigeria, as well as Uganda, Sudan and Democratic Republic of Congo (DRC) in east and central Africa [1]. Between 2014 and 2016, the largest EVD outbreak to date, occurred in West African countries causing death to over 11,000 people and severe economic damage [7]. Uganda and her neighbours (DRC and Sudan) continue to experience frequent EVD outbreaks [6]. In the past two decades, Uganda alone has had six outbreaks in different districts i.e., Gulu (2000), Bundibugyo (2007), Kibaale (2012), Luwero (2011 and 2012) and Kasese ((2019) [8–10]. The first and biggest EVD outbreak registered 425 cases with a case fatality rate (CFR) of 53% [6]. Luweero, an agro-pastoralist district located in the central region of Uganda, experienced two EVD outbreaks between 2011 and 2012 [11]. Considerably, Uganda remains vulnerable to EVD outbreaks particularly in the wake of frequent outbreaks and possible spill over from her neighbours, especially DRC [6].

Besides the enormous impact on human health and health systems, EVD outbreaks affect agricultural production and the food value chain. EVD outbreaks are associated with restrictions in the movement of people and their animals in and out of the affected communities, and disruption of the food value chain, by hindering the transportation/movement of food from farms to consumers [12–14]. The resulting market disruptions lead to below-average food production levels, low purchasing power and reduced incomes, which significantly impact the health and socio-economic wellbeing of families in agro-pastoral communities [15]. Besides, EVD response interventions such as restrictions in movements which affect food security, they are also known to escalate fear, social, political and economic turmoil in the affected and at-risk communities.

Agriculture and particularly, the pig industry in Uganda, is crucial in supporting livelihoods by providing nutritional and income security for 1.1 million smallholder farmers [16]. The country’s rapid growth of the pig population, at present standing at 3.2 million, is attributed to the high financial returns, low cost of production and increased demand for pork [17]. On the whole, urban and peri-urban consumers account for 70% of consumed pork, accessed through the informal local butcheries and pork eateries referred to as ‘pork joints’ [18].

Evidently, the zoonotic epidemics such as EVD cause wide economic impacts beyond the health sector. At present, information on the effect of EVD outbreaks on the pig value chain in Uganda in general and Luwero district in particular is scanty. Likewise, little is known about the knowledge and perceptions of the agro-pastoral communities along the pig value chain in Luwero with regard to EVD outbreaks. This study therefore, assessed the knowledge, perceptions and effects of EVD outbreaks on the pig value chain in Luwero District. In essence, our findings can be used to guide programs aimed to mitigate food insecurity and other consequences associated with EVD outbreaks in agro-pastoral communities that rely on pig production.

## Materials and methods

### Study design

**Fig. 1:**
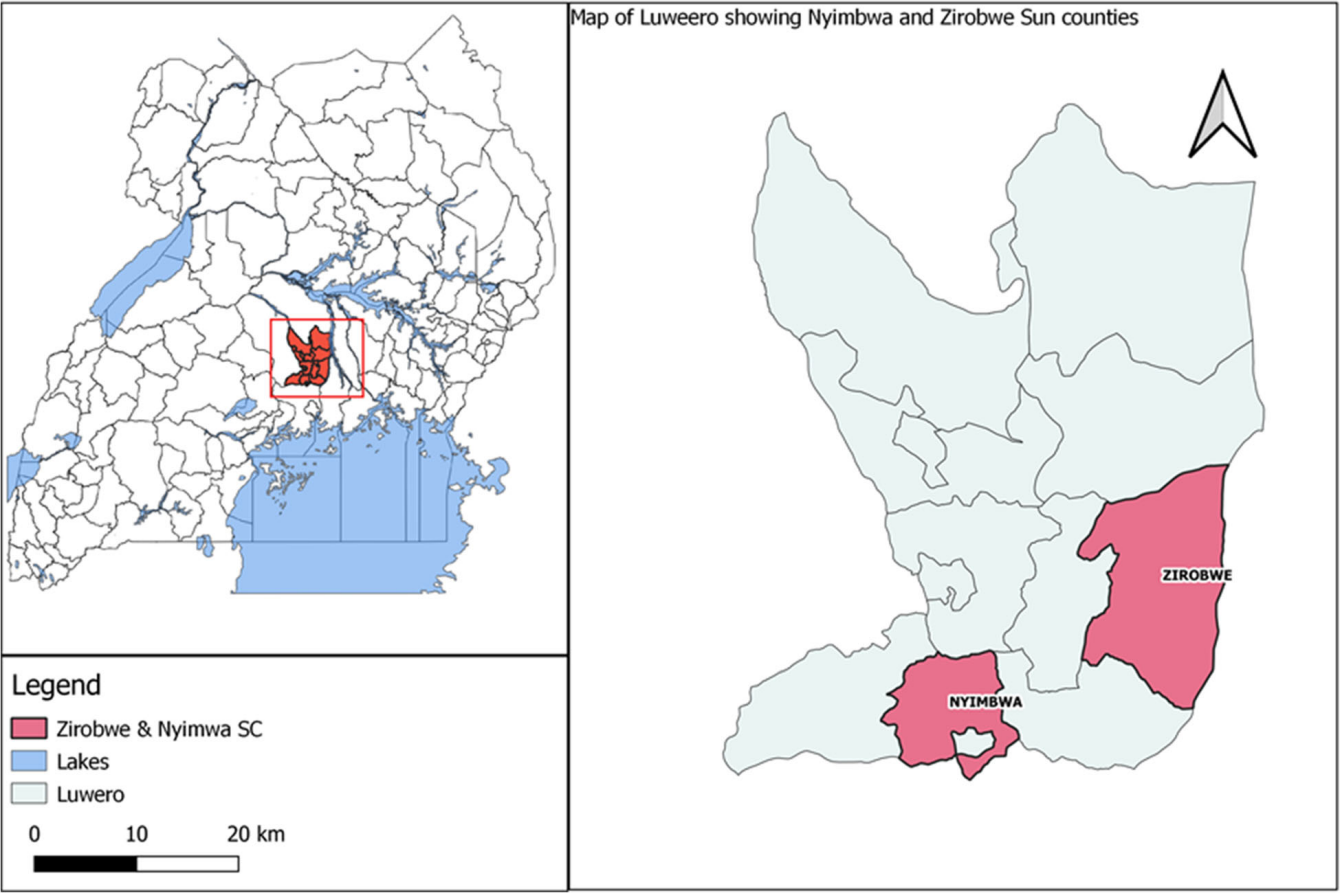
Map of Uganda showing Zirobwe and Nyimbwa sub-counties in Luwero District. Map generated by the research team

This cross-sectional study was conducted in Luwero district, located at 0.8271° N, 32.6277° E in the Central Region of Uganda (Fig. 1**)** from May to July, 2014. Largely, the district’s economic activity is based on rain-fed agriculture and agro-pastoral livestock husbandry practices for 85% of the population. Pig husbandry practiced by small holder farmers is mainly the traditional free range combined with tethering of the animals [19] . The district consists of thirteen sub-counties, of which EVD occurred in Nakisamata village in Ngalonkulu parish, Zirobwe sub-county in May, 2011 and Kakute village, Ssambwe parish, Nyimbwa sub-county in December, 2012, [9, 11].

### Eligibility criteria

The study population involved actors along the pig value chain who fulfilled the eligibility criteria. These were small holder farmers with at least 3 pigs, pig traders involved in sale of pigs, owners of pork-eateries and consumers of pork. Also, households that had registered EVD related illness or death were included in the study. While small holder farmers with 2 pigs or less, had other livestock other than pigs, and respondents that did not consent or did not report EVD related illness/death were not considered for the study.

### Sampling strategy of study respondents

This study used both qualitative and quantitative methods. A structured questionnaire was used to collect quantitative data from individual respondents along the pig value chain. From each parish of Ssambwe and Ngalonkalu, an index villages where EVD outbreak occurred, were purposefully selected. The criteria for selecting the remaining three villages in each parish was basing on a shared common border(s) with the index villages for EVD and having a major road connecting the index to other villages. The adult population (> 18 years) in the two parishes was estimated at 6000 persons with 60% engaged in any form of livestock farming, and 40% (1440 persons) in the pig value chain [17]. A total of 229 respondents was targeted representing about 16% of population actively engaged in the pig value chain. The key aspects considered included respondents’ socio-demographics, level of knowledge on EVD, perception, and effects on pig production, trade and consumption of pork.

The qualitative component utilized focus group discussions (FGDs) and key informant interviews (KIIs) to get an in-depth understanding of the provided data. A total of eight key informants were purposefully selected for the key informant interviews. These were selected based on their experience and knowledge of chronology of EVD outbreaks in study area. The key informants included the community development officer, animal husbandry officers of the respective sub-counties, local council leaders, the district health officer, and village health team members.

A total of four FGDs, two in each parish, composed of 8–10 respondents were conducted among pig farmers, pig traders (butchers and pork eateries) and pork consumers. An FGD guide was used by a moderator in the local language. FGDs were used to explore the knowledge, possible risk factors and effects of EVD along the pig value chain. FGD guides were used to elicit data on perceptions of participants on EVD outbreaks on the pig value chain. FGD participants were purposively selected. FGD participants must have been residents in the two index villages at the time of the EVD outbreak, and must have been engaged in the pig value chain. FGD participants included those engaged in pig rearing, pig trade, butcheries, and pork consumers. We conducted mixed FGDs i.e., comprising of males and females. **M**ixed gender groups tend to improve the quality of discussions and its outcomes [20].

A digital recorder was used to capture the discussions which were later transcribed into English. An informed verbal consent was obtained prior to this. Each session lasted ninety minutes and ended when subsequent discussions produced no new information [21]. Each **FGD was comprised of different categories of respondents which was anticipated to obtain contrasting perspectives that could enrich the discussion.**

### Data management and statistical analysis

Qualitative data from recorded audios was transcribed verbatim and cross-checked to ensure completeness of data by experienced research associates. A thematic content analysis approach was used for analysis of key emergent themes [22].

Quantitative data was entered in Microsoft Excel and exported to SPSS version 22 for statistical analysis. Descriptive analysis was used to summarize demographic characteristics of respondents.

Respondents’ knowledge about EVD was categorised as agree, disagree or I don’t know.

Univariate analysis of categorical data was used in identification of potential risk factors to EVD. The Chi-square test and Fischer’s exact tests were used for cross tabulations. Logistic regression was used to investigate associations between the variables and the outcomes. Variables were considered significant at *p*-value < 0.05. Graph Pad Prism version 6 software was used to carry out repeated measures of analysis of variance (ANOVA) to test the effect on the pig sales at various levels in the pig value chain. Variables were considered significant at *p*-value < 0.05.

## Results

### Respondents’ socio demographic characteristics

Table 1 summarizes the socio-demographic characteristics of 229 respondents that were involved in the study. Slightly more respondents 121 (52.8%) were from Ssambwe parish, almost half 115 (50.2%) were males, most 141(61.6%) attained at least primary level education and majority 184 (80.4%) were farmers. Regarding involvement in pig value chain, most 142 (62%) of the respondents were pig farmers, and few 20 (8.7%) were pig traders.

**Table 1:** Summary of the respondents’ demographic characteristics

		Parish (*N* = 229)		Total
Variable	Attribute	Ngalonkulu *n* = 108	Ssambwe *n* = 121	
Sex	Male	52 (48.1%)	63 (52.1%)	115 (50.2%)
	Female	56 (51.9%)	58 (47.9%)	114 (49.8%)
Household Head	Husband	53 (49.1%)	52 (43.0%)	104 (45.4%)
	Wife	41 (38.0%)	46 (38.0%)	87 (38.4%)
	Child	14 (13.0%)	23 (19.0%)	37 (16.2%)
Level of Education	None	15 (13.9%)	11 (9.1%)	26 (11.4%)
	Primary	71 (65.7%)	70 (57.9%)	141 (61.6%)
	Secondary	22 (20.4%)	37 (30.5%)	59 (25.7%)
	Tertiary	0 (0%)	3 (2.5%)	3 (1.3%)
Occupation	Farmer	97 (89.8%)	87 (71.9%)	184 (80.4%)
	Employed	2 (1.9%)	15 (12.4%)	17 (7.4%)
	Non-employed	2 (1.9%)	6 (5.0%)	8 (3.5%)
	Business (trader)	7 (6.5%)	13 (10.7%)	20 (8.7%)
Role in the pig value chain	Pig farmers	71 (65.7%)	71 (58.7%)	142 (62%)
	Pig traders	7 (6.5%)	13 (10.7%)	20 (8.7%)
	Pork consumers	30 (27.8%)	37 (30.6%)	67 (29.3%)

### Respondents’ knowledge of Ebola virus disease

Respondents were assessed on their ability to recall the occurrence of an Ebola outbreak in the parish or neighbouring village; knowledge of transmission; clinical manifestation and treatment of the disease. Out of the 229 respondents, 219 (95.6%) could recall the occurrence of an Ebola outbreak in their locality (Table 2).

**Table 2:** Respondents knowledge of Ebola virus disease

	Response (%)		
Knowledge assessment	Agree	Disagree	I don’t know
i**) Ability to recall**			
Ability to recall occurrence of Ebola outbreak	219 (95.6%)	8 (3.5%)	2 (0.9%)
**ii) Transmission mode**^a^			
Handling Ebola Infected persons	194 (84.7%)	9 (3.9%)	25 (11.4%)
Eating Bush meat	115 (50.2%)	45 (19%)	69 (30.1%)
Eating monkey meat	148 (64.9%)	15 (6.6%)	65 (28.5%)
Migration of people from Ebola endemic areas	191 (83.4%)	6 (2.6%)	32 (14%)
Public meetings with Ebola infected persons	198 (86.5%)	6 (2.5%)	25 (10.9%)
Eating pork/ touching pigs	56 (24.5%)	169 (73.8%)	4 (1.7%)
iii) **Ebola clinical signs**^a^			
Fever	142 (62.3%)	5 (2.2%)	81 (35.5%)
Vomiting Blood	160 (71.1%)	3 (1.3%)	62 (27.6%)
Diarrhea	171 (75.3%)	3 (1.3%)	53 (23.3%)
Hemorrhage	168 (74.0%)	4 (1.8%)	55 (24.2%)
Muscle Pain	84 (37.0%)	7 (3.1%)	136 (59.9%)
Headache	85 (37.1%)	10 (4.5%)	177 (79.7%)
Skin rash	21 (9.6%)	5 (2.3%)	192 (88.1%)
Sore throat	35 (15.8%)	10 (4.5%)	177 (79.7%)

^a^ Multiple response

On transmission, a majority 194 (84.7%), correctly associated Ebola transmission with handling Ebola infected persons migration of people from endemic areas 191 (83.4%), eating monkey meat 148 (64.9%), eating bats 127 (55.5%) and conducting public meetings where there is an Ebola infected person 198 (64.9%). It was also noted that 56 (24.5%) of respondents associated eating pork or touching pigs to EVD transmission (Table 2).

### Respondents’ knowledge of clinical signs associated with EVD

Regarding clinical manifestation, bleeding from body openings, fever, vomiting blood and diarrhoea were the signs that most respondents highly associated with EVD. However, other signs such as muscle pain, headache, rash and sore throat were not associated with EVD (Table 2)**.**

### Respondents’ perception of pigs as the source of EVD virus disease

The perception about the source or ways through which EVD virus disease can be transmitted was assessed. Of 229 respondents, 56(24.5%) associated occurrence of EVD to eating or touching pigs while 173(75.5%) did not associate EVD virus disease to pigs. Significantly more respondents from Ssambwe (37.2%) than Ngalonkulu (10.2%) associated pigs with EVD in Luwero (*p* < 0.001).

With reference to the perceived risk factors of acquiring EVD virus, there was no correlation between contact with pigs or wild animals and/or their meat products and EVD occurrence in the agro-pastoral community (Table 3). This was also in concurrence with one of the respondents quoted as in the FGD,

**Table 3:** Contact with pigs or wild animals and/or their meat products as perceived risk factor for the EVD in the agro-pastoral community

Variable	Attribute	Presence of Ebola			
		With case (%)	With no cases (%)	Correlation coefficient	p-value
Bush meat consumption	Yes	4 (1.7)	111 (48.5)	−0.005	0.933
	No	4 (1.7)	110 (48.0)		
Consumption of Pork	Yes	7 (3.1)	92 (40.2)	0.105	0.11
	No	1 (0.44)	129 (56.3)		
Monkey meat consumption	Yes	7 (3.1)	142 (62.0)	0.059	0.37
	No	1 (0.44)	79 (34.5)		
Eating Bats	Yes	5 (2.1	124 (54.1)	0.083	0.21
	No	3 (1.3)	97 (42.4)		
Bats in houses	Yes	4 (1.7)	93 (40.6)	0.025	0.703
	No	4 (1.7)	128 (55.9)		
Contact with wild animals	Yes	0 (0.0)	4 (1.7)	−0.022	0.74
	No	8 (3.5)	217 (94.7)		

“.... *… …… … we don’t believe that one can contract Ebola from eating monkey meat, we have eaten bush meat for years and none of us has contracted Ebola.”*

### Effect of EVD outbreak on the demand and sales of pigs and consumption of pork

Out of 142 farmers, 55 (38.7%) believed that EVD outbreaks affected demand of pigs (Table 4). Similarly, from a Focus Group Discussion, a study participant was quoted,

**Table 4:** Effect of EVD outbreak on the pig value chain

Variable	Attribute	Frequency (N) (%)
Reduced Demand (farmers)	Agree	55 (38.7%)
	Disagree	75 (52.8%)
	I don’t know	12 (8.5%)
Reduction in pig sales (Traders)	Yes	17 (85%)
	No	3 (15%)
Consumed Pork during EVD outbreak	Yes	52 (77.6%)
	No	15 (22.4%)

“*… … .. Ebola outbreaks greatly affected demand for pigs and other farm products*. *Traders from Kampala did not come to buy pigs during that period, they feared to acquire Ebola disease.”*

Out of the 20 traders, 17 (85%) were in agreement that EVD reduced pig sales (Table 4). There was a significant reduction (*p* < 0.001) in pork consumption during EVD outbreaks. Out of the 67 consumers in this study, 15 (22.4%) did not consume pork during the EVD outbreak (Table 4).

In concurrence with FGD, one respondent was quoted as,

“… … *I feared to consume pork because I thought I would contract Ebola disease; we were told to avoid consuming any meat during that period*.”

As in Table 5, there was a significant reduction (*P* = 0.001) in the average number of pigs sold during EVD outbreak period. There was also a significant reduction (*P* = 0.04) in the average number of pigs bought by traders during the EVD outbreak. Similarly, it is clearly seen that the number of pigs sold/ slaughtered by butcher men at pork eating places reduced significantly (*P* = 0.03).

**Table 5:** Pig sales and price indices during EVD outbreak period in Luwero district

	Mean SD (Pigs sold per period)			
Persons involved in pig sales	Before	During	After	***P***-value
Farmer *N* = 142	4 ± 3	3 ± 1	3 ± 2	0.001*
Trader *N* = 20	4 ± 3	4 ± 2	5 ± 3	0.04*
Pork butchers *N* = 9	7 ± 3	5 ± 3	6 ± 3	0.03*
**Price indices**	**Estimated average kg SD)**	**Price per Kg (USD**	**USD**	
Price	426 + 255	353 + 237	326 + 250	
Price/kg (US$)	2.5	1.6	1.8	
Total US$	1065.00**	564.8*	684.00**	

*Significantly lower during the EVD outbreak (Repeated measures ANOVA, *P* < 0.05)

**statistically significant when compared before, during, and after EVD outbreak

The unit cost of a kilogram of pork reduced during the outbreak. The income generated from pork sales reduced significantly during the outbreak when compared to the period before and after the outbreak (Table 5).

## Discussion

This study assessed the perceptions and knowledge of occurrence of EVD, and its effects on the pig value chain in an agro-pastoral district in central Uganda. With regards to knowledge, a majority of the respondents were able to recall previous EVD outbreaks in Luwero that occurred more than two years ago. This was contrary to common health care events, often not recalled accurately within a few months of occurrence [23]. Most likely, the impact of EVD outbreaks associated with economic turbulence and social stigma in communities appear to last longer.

The fact that the agro-pastoral community respondents were able to identify the major signs and symptoms of EVD such as fever, vomiting, bleeding and diarrhoea was not unexpected. These were frequently shared on the information education and communication materials that were available in the community health centres and public spaces in Luwero [24]. The community can ably avoid the EVD infections when such cases reoccur. However, other important signs which are frequently associated with common ailments such as malaria could not be identified, suggesting that the community could mistakenly handle EVD patients with minimal or no precautionary measures. This is particularly possible if EVD presents with nonspecific symptoms that can easily be mistaken for other endemic diseases like malaria, typhoid fever, yellow fever or, even measles in children [25]. Efforts should be put in place to educate the public to consider patients with such signs as suspected cases, especially when there is an EVD outbreak in the community.

A majority of the respondents linked the transmission of EVD with eating of bats, monkey and bush meat. This was in conformity with the previous studies [5, 26, 27], which noted that consumption of bush meat could contribute to infection. Conversely, the respondents did not consider eating of game meat as a high risk factor, probably because in earlier EVD outbreaks that occurred in Kibaale district [28] and also in Luwero district [9] the victims could not be linked to eating of wild animals such as monkeys before falling ill. Meanwhile, as eating of bats or other wild animals could not be regarded as a high risk for acquiring EVD, hunting and eating of game meat continues unabated, exposing the vulnerable communities to future EVD infections. Besides hunting game meat for authentic values, most communities depend on wild game as source food and income security. Efforts to provide alternative livelihoods and food security could protect vulnerable communities from EVD.

Our findings showed that the unit cost of pork was significantly reduced during EVD outbreak. Also, the average number of kilograms of pork sold as well as the final income significantly reduced during the outbreak. This was in conformity with the assertions that beyond the public health impacts, emerging global infectious cause wider socioeconomic consequences often disregarded in impact assessments [29]. This could be attributed to the human health sector-based EVD preparedness and prevention practices and the public behaviour of stigmatizing the pigs as the source of EVD that led to avoidance of eating pork. Evidently, zoonotic infectious disease outbreaks have been associated with substantial economic impacts due to disruption of the livestock markets [29]. In consequence, the reduced sales and incomes negatively impacted the household incomes. In accord with prior reports from West Africa, household and community incomes dropped significantly during the EVD outbreak [30] leading to increased food insecurity and household vulnerability.

A plausible explanation for the reduced sales of pigs/pork was due to the misconception that pigs were the source of EVD, creating fear of contracting the disease and avoiding eating of pork by the consumers. It was not clear why the community associated pigs with the EVD. However, the two EVD outbreaks in Luwero were apparently preceded by African swine fever (ASF) epidemics with wide spread mortalities of pigs in the community. Besides, pigs with ASF show signs of haemorrhages on the skin and in internal organs, labored breathing with frothy nasal discharges mixed with blood, and death of most affected animals within 2-5 days [31]. Farmers have frequently observed reddening of the ears, clinical signs consistent with of ASF [32, 33]; but more less the same sign (bleeding) is observed in human cases with EVD. As a result, the ASF in pigs and EVD in humans are erroneously believed to be caused by the same agent that originates from pigs. To clear this misconception, a deliberate social-anthropological understanding of the agro-pastoral communities with regard to such diseases should be given priority attention to minimize losses to the pig industry in future.

Our focus on the EVD outbreak with respect to the pig value chain, was prompted by Uganda’s fast growing pig sector, that continues to register increasing pork consumption rates (average of 3.4 kg/person per year), the highest in East Africa [34]. Primarily, the national EVD control programs are designed to protect the human health and remotely consider enterprises that support farmers’ livelihoods. However, the neglect of the community’s survival and sustenance options, especially the pig value chain could lead to adversative effects on livelihoods. Effective post-EVD intervention plans in Uganda and elsewhere, must encompass the primary sources of livelihoods for such agro-pastoral communities in order to mitigate the negative impacts of the disease. For instance, farmers could be provided with breeding pigs, access to credit, and training for the small holder pig enterprises. This is in concurrence with strategies put in place in West Africa in lieu of efforts to stimulate social and economic recovery of vulnerable communities affected by EVD [35]. EVD control programs should be cognisant of the inter-sectoral collaboration that would assure sustainable pig production, supply and marketing for the small holder pig farmers in Uganda.

## Conclusion

There was a significant reduction in pig sales, price per unit cost of a kilogram of pork and pork consumption. The perceived association of EVD with pigs is detrimental to the pig industry and could affect the entire pig value chain in terms of pig production, pig sales and pork consumption.

### Recommendations

There is a need for improved health education and messaging before and during times of outbreaks to reduce the misconceptions and ensure that the agro-pastoral community is informed of the real risks for being infected by EVD and proper health prevention related to contact with domestic or wild animals and/or their meat products. This should involve collaboration among the sectors of agricultural, health and wildlife so as to design appropriate EVD response programs for the agro-pastoral community in Uganda.

## Supplementary Information

**Additional file 1.** Survey Questionnaire tool.

**Additional file 2.** Interview guiding questions for Focus Group Discussions.

## Abbreviations

ANOVA: Analysis of Variance
ASF: African Swine Fever
CFR: Case Fatality Rate
EVD: Ebola virus disease
FGD: Focus Group Discussion
KII: Key Informant Interview
