# Supplementary material for: Knowledge, perceptions and effects of Ebola virus disease outbreak on the pig value chain in the agro-pastoralist district of Luwero, Central Uganda
Source: BMC Infect Dis. 2021 Jul 9;21:669. doi: 10.1186/s12879-021-06337-8 (PMC8268591; doi:10.1186/s12879-021-06337-8)
Supplement: Supplementary file 1 — Additional file 1. Survey Questionnaire tool. [file 12879_2021_6337_MOESM1_ESM.docx]

**Supplementary file 1:** **Survey Questionnaire tool**

**RISK FACTORS AND EFFECT OF EBOLA OUTBREAKS ALONG THE PIG VALUE CHAIN IN NYIMBWA AND ZIROBWE SUBCOUNTIES IN LUWERO DISTRICT**

**Introduction**

We are carrying out a research on the Effect and Risk factors of Ebola Outbreak on the pig value chain in this district. It has been discovered that pigs can be infected with Ebola Hemorrhagic fever and so this study aims at identifying the risk factors and losses of Ebola to humans along the pig value chain. We would like to inform you that there is no risk when you participate in the study and all the information will be kept confidential.

Do you agree to participate in this study?

Yes……………………………………. No……………………………………….

Date………………………………………………..

1. Id of the respondent: ………………………………

2. Gender……………………………………………….

3. Age……………………………………………………

**Geographical Data**

**4.** Nyimbwa sub county Zirobwe Sub County

**5**. Local council 1(Village) _______________________

1. **HOUSEHOLD COMPOSITION**

| 2.1 | Status of Respondent | Husband……………………………………………….…...1  Wife………………………………..………………….........2  Child…………….………………..…………………..……..3  Others(Specify)…………………………………………….4 |
| --- | --- | --- |
| 2.2 | Religion | Christian…………………………………………….…..…1  Muslim……………………………….……………………2  Traditional……………………………….…………..…..…3  Others(Specify)……………………….……...……..……..4 |

| **2.3** | Education level of Respondent | None…………………………………………………………..1  Primary………………………………………………………..2  Secondary……………………………………………………..3  Tertiary………………………………………………………..4 |
| --- | --- | --- |
| **2.4** | Main Occupation of the respondent | Farmer……………………………………………………...…1  Employed……………………………………………………..2  Non- Employed…………… ……………………….......…….3  Business(trader)……………………………………………….4 |

**3. KNOWLEDGE ABOUT EBOLA VIRAL HEMORRHAGIC FEVER**

| 3.1 | Do you know what Ebola is? | Yes………………………………………………1  No……………………………………………….2 | | |
| --- | --- | --- | --- | --- |
| 3.2 | If yes, Have you ever heard about its occurrence in your neighborhood? | Agree……………………………………………1  Disagree…………………………………………2  I don’t know……………………………...……..3 | | |
| 3.3 | Which year did it occur? | 2011…………………………………….………1  2012…………………………………….………2  2013………………………………….…………3  Doesn’t know…………………….…..………..4 | | |
| 3.4 | How long did the Ebola outbreak last? | 1 month………….………………..………..…..1  2 month…………………………..………..…...2  3 months…..……………………………………3  4 months & above…………………....………...4  Doesn’t remember…………………..…..……...5 | | |
| 3.5 | **What are some of the ways through which Ebola can be transmitted?** | Agree (1) | Disagree (2) | I don’t Know (3) |
|  | i)handling Ebola Infected persons |  |  |  |
|  | ii) Eating Bush meat |  |  |  |
|  | iii) Eating pork/ touching pigs |  |  |  |
|  | iv) Migration of people from endemic areas |  |  |  |
|  | v) Public Meetings |  |  |  |
|  | vi) Eating monkey meat |  |  |  |
|  | vii) Eating Bats |  |  |  |
|  | viii) Which are the other ways |  | | |
| 3.6 | Have you ever seen any Ebola patient | Yes……………………………………………….1  No……..………………………………….………2 | | |
| 3.7 | **If yes, which signs of Ebola Hemorrhagic fever do human beings present** | Agree (1) | Disagree(2) | I don’t Know(3) |
|  | a)Fever |  |  |  |
|  | b) Intense weakness |  |  |  |
|  | c) Muscle pain |  |  |  |
|  | d) Headache |  |  |  |
|  | e) Sore throat |  |  |  |
|  | f) Vomiting blood |  |  |  |
|  | g) Diarrhea |  |  |  |
|  | h) Rash |  |  |  |
|  | i) Bleeding from all body openings |  |  |  |
|  | j) Excessive sweating |  |  |  |
|  | k) Chest Pain |  |  |  |
|  | Do you think Ebola can be treated? | Yes……………………………………………..…1  No…………………………………………..….…2  I don’t know…………………………………..….3 | | |
| 3.6 | **If yes, what are some of the ways through which Ebola can be treated** | Agree (1) | Disagree (2) | I don’t know(3) |
|  | Traditional healer |  |  |  |
|  | Doctor at clinic/hospital |  |  |  |
|  | Pharmacist/drugstore |  |  |  |
|  | Herbs/local remedies |  |  |  |
|  | Village health Teams |  |  |  |
|  | No treatment |  |  |  |
|  | Others (Specify) |  |  |  |

**4. EFFECTS OF EBOLA OUTBREAKS ON PIG PRODUCTION (To the Farmer)**

| 4.1 | Do you rear pigs? | Yes…………………………………….…..1  No……………………………………….…2 |
| --- | --- | --- |
| 4.2 | If yes, where do you buy your pigs from? | Nearby Market……………………………..1  Farms………………………………………2  Neighboring village…………………..……3  Research institutions………………………4  Rearing for a friend………………………..5 |
| 4.3 | If no, have you ever worked in a pig enterprise? | Yes…………………………….…………..1  No…………………………………………2 |
| 4.4 | Which farming system is carried out on your farm? | Intensive…….……………………………..1  Semi- intensive…………………………….2  Tethering……………………………………3  Free range system………………………..4 |
| 4.5 | Do you think pigs are a source of Ebola to humans? | Agree………………..……………………..1  Disagree……………..……………………..2  I don’t know……….………………………3 |
| 4.6 | What is your herd size? | Piglets |
|  |  | Adults |
| 4.7 | Did the herd size increase during Ebola period? | Agree……………………………………….1  Disagree…………………………………….2  I don’t Know……………………..…….…..3  Constant…………………………………….4 |
| 4.8 | What was the reason for the increase? | Reproduced…………………………………1  Bought others……………………………….2  Care/ Medication……………………………3  Didn’t want to sell………………………….4  Reduced demand…………………………5 |
| 4.9 | Did the herd size decrease during Ebola Outbreak period? | Agree………………………………….…1  Disagree………………………………….2  I don’t know……………………………..3 |
| 4.10 | What was the reason for the decrease? | Death…………..……………………………1  Sold…………………………………………2 |
| 4.11 | Pig Sales were made during the Ebola outbreak? | Agree…………………………………….…1  Disagree…………………………………….2  I don’t know………..………………………3 |
| 4.12 | Pig Sales increased during the Ebola outbreak period | Agree…………………………………….…1  Disagree………………………………….…2  I don’t know…………………………..…...3  No change………………………………….4 |
| 4.13 | What was the reason for their increase? | Increased Demand…………………………1  Sold …………………………….…………2  Not associated……………………………..3  Reproduced………………………………..4 |
| 4.14 | Pig Sales decreased during the Ebola outbreak period | Agree……………………………………….1  Disagree…………………………………….2  I don’t know…………………………….….3  Constant……………………………………4 |
| 4.15 | What was the reason for the decrease in pig sales? | Below maturity…………………………….1  Reduced demand…………………………..2  Fear…………………………………………3  Death………………………………………..4  Slaughtered them……...……………………5  I don’t know………………………………..6 |
| 4.16 | What used to be the average number of pigs sold per month before the occurrence of Ebola outbreak? |  |
| 4.17 | What was the number of pigs sold per month during the outbreak? |  |
| 4.18 | What was the number of pigs sold per month after the outbreak? |  |
| 4.19 | After the area was declared Ebola free, how long did it take your sales to go back to the average sales? | 1 month………………………………….….1  2 months…………………………………….2  3 months…………………………………….3  4 months & above…………………………..4 |
| 4.20 | What is the number of pigs sold per month currently? |  |
| 4.21 | Where do you sell your pigs? | Directly to consumers…………………..…1  To middle men………………….……..…..2  To butcher men…………………………....3  To middle men and butcher men………….4  To farmers…………………………...….5 |
| 4.22 | Ebola outbreak affected demand for pigs | Agree…………………….…………………..1  Disagree………….…………………………..2  I don’t know…………………………………3 |

**5. EFFECT OF EBOLA ON PIG/ PORK SALE OUTLETS (Pig trader and Butcher)**

| 5.1 | Pigs are source of Ebola to humans | Agree…………………..…….……….…..1  Disagree…………….……………………2  I don’t Know…………….…..………….….3 |
| --- | --- | --- |
| 5.2 | Markets were closed during Ebola outbreak period | Agree…………………..………….....…..1  Disagree……………………………..……2  I don’t know………………………..…….3 |
| 5.3 | Markets were closed for a period of: | A month…………………………………..1  A week…………………………..………2  2- 3 weeks……………………...…………3  1-3 days……………………………………4 |
| 5.4 | Why do you think markets were closed during the Ebola outbreak? | Avoid spread……………………………...1  Fear……………………………………….2 |
| 5.5 | Did market closure due to Ebola outbreaks affect the pig sales? | Yes………………………………….………1  No………………………………….……..2 |
| 5.6 | If yes, what was the effect? | Increased sales…………….……………..1  Decreased sales………………………...2  No change…………………………….…..3 |
| 5.7 | How many pigs did you use to sell before the outbreak per month? |  |
| 5.8 | How many pigs were you selling per month during the outbreak? |  |
| 5.9 | How many pigs are you selling per month currently? |  |
| 5.10 | Do you think pork consumers associated Ebola outbreaks with piggery/ pork handling? | Agree………………………….………...1  Disagree…………..….………….……….2  I don’t know……………………………3 |
| 5.11 | What were the changes in pork consumption during the outbreak? | Decreased…………………………….….1  Increased…………………………………..2  No change…….………………………..…3  Others(specify)…………………….…..4 |
| 5.12 | What number of pigs did use to buy per month before the occurrence of Ebola outbreak? |  |
| 5.13 | What number of pigs did you buy per month during the outbreak? |  |
| 5.14 | Number of pigs bought per month after the outbreak? |  |

**6. KNOWLEDGE, ATTITUDE OF PORK CONSUMPTION DURING EBOLA OUTBREAKS (pork Joint Owners)**

| **6.1** | Pork consumption increased during Ebola outbreak period | Agree…………………..………..….1  Disagree………………….…………2  I don’t know……….…….…………3  No change…………………………..4 |
| --- | --- | --- |
| **6.2** | Pork consumption decreased during Ebola outbreak period | Agree………………………….…….1  Disagree…………….……….………2  I don’t know………………………..3 |
| **6.3** | There is a possible linkage between Ebola outbreaks and Pigs/ pork consumption | Agree………………………….….…1  Disagree……………………….…….2  I don’t know………………….……..3 |
| **6.4** | Do you consume pork? | Yes…………………………………..1  No…………………………..………..2 |
| **6.5** | Did you consume pork during the Ebola outbreak | Yes……………………………..…….1  No……………………………..……..2 |
| **6.6** | If no, what were the reasons for not consuming pork during the Ebola outbreak period | Fear…………………………………1 |

Thank you very much for allowing me to have a discussion with you.
