# Supplementary material for: Knowledge, perceptions and effects of Ebola virus disease outbreak on the pig value chain in the agro-pastoralist district of Luwero, Central Uganda
Source: BMC Infect Dis. 2021 Jul 9;21:669. doi: 10.1186/s12879-021-06337-8 (PMC8268591; doi:10.1186/s12879-021-06337-8)
Supplement: Supplementary file 2 — Additional file 2. Interview guiding questions for Focus Group Discussions. [file 12879_2021_6337_MOESM2_ESM.docx]

**Supplementary file 2: Interview guiding questions for Focus Group Discussions**

**RISK FACTORS AND EFFECT OF EBOLA OUTBREAKS ALONG THE PIG VALUE CHAIN IN NYIMBWA AND ZIROBWE SUBCOUNTIES IN LUWERO DISTRICT**

**Introduction**

We are carrying out a research on the Effect and Risk factors of Ebola Outbreak on the pig value chain in this district. It has been discovered that pigs can be infected with Ebola Hemorrhagic fever and so this study aims at identifying the risk factors and losses of Ebola to humans along the pig value chain. We would like to inform you that there is no risk when you participate in the study and all the information will be kept confidential.

Date……………………………. No. of Discussants……………………………………

FGD composition: Male……………………………Female……………………………….

Time start…………………………………………. Time end……………………………..

Location of the discussion………………………………………………………………….

**Consent Note**:

We are from Makerere University, College of Veterinary Medicine Animal resources and Biosecurity. We are here to learn from you about risk factors and effects of Ebola outbreaks on the pig value chain. The information generated will help in Ebola risk management

Can we proceed? Yes…………………………… No……………………………………..

1. Has an Ebola outbreak ever occurred in this village / neighboring village?

2. Which year did it occur?

3. How long did the Ebola outbreak last?

4. What are the possible causes of Ebola outbreaks?

5. Did you see/ hear about Ebola patients?

6. What are the signs of Ebola Hemorrhagic fever in humans?

7. Were the Ebola patients treated?

8. What treatment was given to the Ebola patients?

9. Do you know of any pig farmers in this village?

10. Do people here associate pigs and Ebola Hemorrhagic fever in humans?

11. What are some of the activities that these farmers carry out and can predispose them to Ebola Hemorrhagic fever?

12. Do you think Ebola can be transmitted through handling Pigs?

13. How did Ebola outbreaks affect pig production in this area?

14. What are some of losses that were incurred along the pig value chain during Ebola outbreaks?

15. How regularly do you consume pork?

16. How much do you spend on pork consumption per month?

17. What is your view on the pigs as a possible source of Ebola to humans?

18. What has changed on pork consumption during the Ebola outbreak?

19. What were the reasons for not consuming pork during the Ebola Outbreak period?
